# Acupuncture and botulinum toxin A injection in the treatment of chronic migraine: A randomized controlled study

**DOI:** 10.22088/cjim.8.3.196

**Published:** 2017

**Authors:** Bahram Naderinabi, Alia Saberi, Masood Hashemi, Mohammad Haghighi, Gelareh Biazar, Farid Abolhasan Gharehdaghi, Abbas Sedighinejad, Tahereh Chavoshi

**Affiliations:** 1 Department of Anesthesiology, Anesthesiology Research Center, Guilan University of Medical Sciences, Rasht, Iran.; 2 Neurosciences Research Center, Neurology Department, Poursina Hospital, School of Medicine, Guilan University of Medical Sciences, Rasht, Iran.; 3 Anesthesiology Department, Shahid Beheshti University of Medical Sciences, Tehran, Iran.; 4 Pain Department and Research Center, Mahak Pediatric Cancer Treatment and Research Center, Tehran, Iran.

**Keywords:** Migraine, Chronic, Acupuncture, Botulinum Toxin-A, Pain Management

## Abstract

**Background:**

Migraine is a common type of headache. Sometimes adequate pain relief is not achieved by conventional treatments. Acupuncture and botulinum toxin-A injection are known as non-pharmacological interventions for this purpose. The aim of this research was to compare the effect of acupuncture with botulinum toxin-A injection and pharmacological treatment in controlling chronic migraine.

**Methods:**

This clinical trial was conducted on patients with chronic migraine in the North of Iran during 2014-2015. Eligible patients were randomly allocated to groups receiving acupuncture (A) or botulinum toxin A (B) and controls (C) by designed quadripartite blocks. All patients were evaluated at baseline, one, two and three months after treatment using visual analogue scale (VAS) score and other parameters. The analysis of data was performed in SPSS software Version 19.

**Results:**

One hundred fifty patients (48 males and 102 females) completed this study. During the 3- month study, the pain severity significantly diminished in three groups (P=0.0001), with greater reduction in group A (P=0.0001). The number of days per month with migraine, absence from work and the need for medication significantly decreased in three groups at 3 times of evaluation (p<0.05) with fewer side effects in group A (P=0.021).

**Conclusion:**

Acupuncture, botulinum toxin-A injection and pharmacological treatment have beneficial effects on chronic migraine; however, acupuncture showed more effectiveness and fewer complications.

**M**igraine is a primary headache disorder. It approximately affects fourteen percent of the general population with a higher incidence in women (1, 2). Migraine is reported as a debilitating condition which results in economic burden and impairs the quality of life of the patients. It is especially accurate in chronic migraine, previously referred to as transformed migraine included in the International Classification of Headache Disorders 3rd edition (ICHD-3 beta version), with code 1.3. It is characterized by headaches (tension-type like and/or migraine) on 15 or more days per month, with at least 8 days per month fulfilling International Headache Society diagnostic criteria for migraine for at least 3 months in the absence of ongoing medication overuse (3). There are some associated conditions in chronic migraine with hopes of getting rid of the chronicity via prevention (4-7). Also, there are several chemical and synthetic treatments for pain relief especially chronic head pain (8, 9). However, their poor efficacy may result in unacceptable side effects and overdose. 

Besides, it may lead to distress and pain in many patients. Although there are some natural treatments for migraine including omega 3 (10), it may be ineffective in chronic migraine. Therefore, it is vital to investigate new non–pharmacological modalities accompanied with less adverse effects and more efficiency (2, 11-13). At present, in clinical practice, botulinum toxin type A and acupuncture are currently applied as non-pharmacological therapy choices in headache care. Acupuncture, as a part of the Traditional Chinese Medicine (TCM) describes group of procedures that produce clinical effects with a variety of techniques. Neural imaging studies have demonstrated that the endogenous opioid system and the central nervous system play an important role in acupuncture analgesia. Positron emission tomography scans demonstrate that acupuncture causes short and long term increases in limbic system mu-opioid-binding potential. It results in the release of endorphins and facilitation of spinal cord pain processing inhibition and anti-inflammatory action. Several large comparative studies have shown that acupuncture is more effective or equal to standard treatment of migraine (13-16). 

Moreover, botulinum toxin-A synthesized from gram-positive anaerobic clostridium botulinum was approved by the Food and Drug Administration (FDA) to prevent migraine in 2010 (2, 17). It has also been used for other kinds of neuromuscular and pain disorders (18, 19). The mechanism of action inhibits the peripheral release of acetylcholine at neuromuscular junction leading to prolonged muscle relaxation (2, 20, 21). In addition to blockage of acetylcholine release, it inhibits the release of numerous neurotransmitters and neuropeptides such as neuropeptide-Y, substance P, calcitonin gene-related peptide (CGRP) which are involved in the mechanism of pain. CGRP causes the release of pro-inflammatory mediators in correspondence with the duration of a typical migraine episode. Botulinum toxin has no direct effect on the central nervous system as it cannot penetrate the blood brain barrier. Yet, it reaches the central nervous system (CNS) by retrograde axonal transport, but as this movement is so slow, it seems likely to be inactivated before reaching the CNS. Some indirect effects of botulinum toxin have also been identified in the CNS. On the spinal level, botulinum toxin is capable of producing reflex inhibition of the muscle spindle organ. On the supraspinal level, studies have demonstrated that botulinum toxin can normalize the altered intracranial inhibition and altered somatosensory evoked potentials. Botulinum toxin inhibits the release of neurotransmitters for nociceptive nerve terminal and exerts an analgesic effect. Major effects appear five to six weeks after the intramuscular injection and recovery which means regeneration of the never endings occurs after twelve weeks. Therefore, injection cycles should be repeated every three months (2, 21, 22).

There is now increasing evidence documenting the therapeutic effects of these two methods. The aim of the present study was to compare the efficacy of botulinum toxin type A with acupuncture and medical treatment (as control) in patients with chronic migraine. Change in pain severity, medication usage, the number of pain days in a month, the number of days per month which medication is needed have been compared in this study between different treatment groups. Besides, the adverse effects of those treatments have been determined and compared. 

## Methods

In this clinical trial, two hundredthirty patients with chronic migraine were enrolled in Guilan Pain Clinic, North of Iran, from March 2014 to February 2015. This study was registered in Iranian Registry of Clinical Trial (IRCT) with ID number of 201404146186N3. Inclusion criteria: Patients suffering from chronic migraine diagnosed based on the criteria of the International Classification of Headache Disorders 3rd edition (ICHD-3 beta version) established by a neurologist, aged between 20-60 years, normal liver function and coagulation tests.

Exclusion criteria: Intolerable side effect occurrence, concomitant medication overuse headache and other types of headache based on the abovementioned diagnostic criteria, opioid abuse, recent use of prophylactic drugs (including ß blockers, sodium valproate, tricyclic antidepressants, topiramate, flunarizine and any other formulated prophylactic medications) in the last three months, other present or past neurologic disorders including epilepsy, multiple sclerosis, neuropathy and myopathy, myofascial pain syndrome established by history examination and/or documented paraclinical tests, past history of receiving acupuncture and botulinum toxin A, pregnancy and lactation.

Participants: Two hundred thirty patients suffering from chronic migraine enrolled in this study. To assure their physical and mental health, a medical history and routine laboratory tests were obtained (cell blood count, liver function tests and prothrombin time and partial thromboplastin time). After assessing eligibility considering inclusion and exclusion criteria, a total of 162 patients remained; who were randomly allocated to acupuncture group (A), botulinum toxin-A group (B) and control group (C) by designed quadripartite blocks. Our subjects had an equal probability of being assigned to any of the three groups.

Before the treatment, an informed consent was obtained from all patients. The principles of the Declaration of Helsinki (1964) have been taken into consideration in this research. Our patients were interviewed by a neurologist at Guilan Pain Clinic. This interview included the following items: migraine history, attack frequency and detailed description of the migraine pain. All patients were allowed to treat their acute migraine attacks with Novafen (Alhavi Pharmaceutical Company). In addition, drug dose and number of days of medication should be recorded.

The style of acupuncture in traditional chinese medicine (TCM) was considered in group A. The procedure was performed by a fixed experienced acupuncturist. First, the skin was disinfected with around 75% alcohol. The acupoints are located in palpable depressions in the body surface between muscles, tendons or bones. Based on TCM practice, the acupuncture points used were mainly the gallbladder (GB) 41, GB 20, GB 15, GB14, GB10, GB8, large intestine (LI) 4, liver 3, Sanjiao 5, Du-Mai 20, and Extra 2 Taiyang alone with points for individual migraine associated symptoms. Needles were inserted to a depth of 10-15 mm and often bilateral. 10 -12 disposable sterile needles in two sizes gauge 32 length of 25mm and 40mm were used. The needles were inserted and manual manipulation, lifting, thrusting and rotating were continued until deqi sensation from needling was experienced by the patient. This sensation might be reported as numbness (A-beta fiber activation) or as aching, dull, heavy and warm sensation (A-delta or C fiber activation). Our subjects received thirty treatment sessions of aquavit on selected points in sixty days in two cycles with one week rest between two cycles. During the procedure, our subjects were comfortably seated at a temperature of 24˚C and had one hour of acclimatization before the intervention was started (23).

While in group B, we followed the protocol of the phase ΙΙΙ Research Evaluating Migraine Prophylaxis Therapy I (PREEMPTI). Furthermore, botulinum toxin A was injected to patients by the same experienced acupuncturist. Injection was done in thirty one trigger zones over the facial and pericranial muscles, at the total dosage of 155 U. 1cc tuberculin syringe with 0.01 gradations and a long 30 g needle was employed for the procedure. Afterwards, we used ice packs to provide cooling effect for skin before and after injection to prevent solution diffusion. Massaging of the site was avoided. To provide more control of the needle, injection was preformed while advancing the needle (17). 

As for control (C) group, the patients have received sodium valproate 500 mg/day for three months. A designed checklist containing the following items was filled by a physician who was blinded to the type of treatment at baseline (T0), one month (T1), two months (T2) and three months (T3) after treatment: adverse effects, pain severity, decreased medication use, and the number of days per month of pain the number of days per month with the need of medication and days missed out due to headache. Assessing pain intensity was based on visual analogue scale (VAS) score.

Statistical analysis: For the analysis of data, first the normality of parameters was assessed by Kolmogorov Smirnov (KS) test and then parametric (two independent sample t-tests) or nonparametric Mann-Whitney U tests were used according to their normality. The other analysis was done by chi-square test, Cochran's Q test, general linear model and repeated measure ANOVA to analyze and compare the studied parameters in each group. Unpaired t-test and chi-square test were used to compare the categorical variables between two groups. The data were expressed as mean ± standard deviation (SD). A p<0.05 was considered statistically significant. All the statistical analysis was performed using the SPSS statistical software Version 19 (SPSS Inc. Chicago II). 

## Results

From two hundred thirty patients who referred to our clinic, one hundred sixty-two subjects who met the inclusion criteria enrolled the study. A total of 150 patients (50 in each group) completed the survey while the other participants dropped out because of low compliance but not affected by severe adverse effects. The mean age of the subjects in groups B, A and C was 36.8±7.4 and 37.2±7.3 and 37.6±7.4 years, respectively (P=0.876). In group A, B and C, 24 (58%), 27 (54%) and 33 (66%) respectively were women and the others were men (P=0.461). The mean duration of migraine in groups A, B and C was 10.3±5.5, 9.2±5.3 and 9.1±3.9 years, respectively (P=0.407) and they had taken migraine prophylaxis for the period of 4.2±3.6 years, 3.2±3.2 and 4.1±2.4 years, respectively (P=0.224). The demographic data of patients, primary headache characteristics and associated symptoms in the three groups have been reported in table 1.

**Table 1:** Demographic data of patients, primary headache characteristics and its associated symptoms in three treatment groups

**p-value**	**Sodium Valproate**	**Acupuncture**	**Botulinum toxin-A**		
0.876	37.6±7.4	37.2±7.3	36.8±7.4	Age	
0.461	17(33%)	21(42%)	23(46%)	Male	Gender
	33(66%)	29(58%)	27(54%)	Female	
0.407	9.15±3.95	10.34±5.46	9.22±5.31	Duration of migraine (year)	
0.224	4.11±2.45	4.22±3.57	3.23±3.19	Duration of drug use (year)	
0.072	21.02±4.36	21.26±6.84	23.56±6.46	Day/month headache	
0.060	14.1±5.06	14.56±5.62	17.76±6.18	Number of drug use/month	
0.120	8.36±1.39	8.56±1.29	8.9±1.24	Headache severity (VAS* score)	
0.443	45%	48%	45%	Absence from work (% of patients)	
0.378	31(62%)	36(72%)	37(74%)	Photophobia	
0.699	27(54%)	30(60%)	31(62%)	Phonophobia	
0.707	26(52%)	27(54%)	23(46%)	Osmophobia	
0.809	32(64%)	34(68%)	35(70%)	Pulsating	
0.434	38(76%)	41(82%)	43(86%)	Nausea &Vomiting	

* Visual Analog Scale

**Figure 1:**
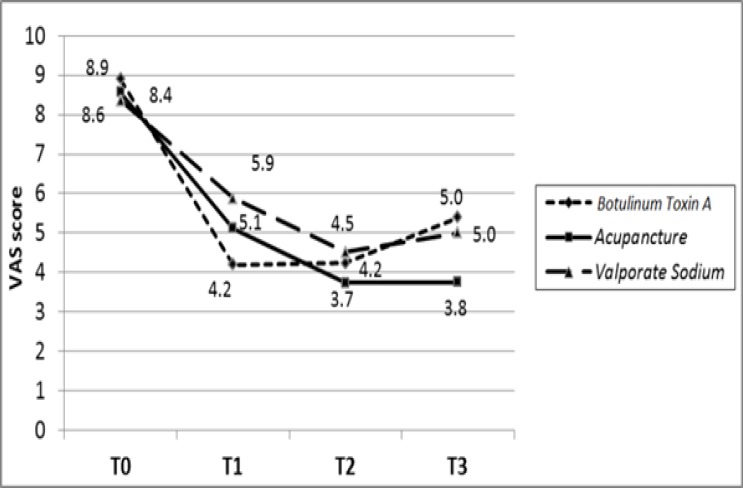
The trend of changes of mean VAS score in groups treated by acupuncture (A) (P=0.0001), botulinum toxin A (B) (P=0.0001) and valproate sodium (P=0.0001)

VAS score significantly diminished from baseline to T3 in three groups (p=0.001). Comparing within the groups, group A significantly showed more reduction in VAS score (P=0.0001). There were no difference of VAS score at baseline (P=0.12), but were significant at T1 (P=0.0001), T2 (P=0.001) and T3 (P=0.0001) (figure 1).

**Figure 2:**
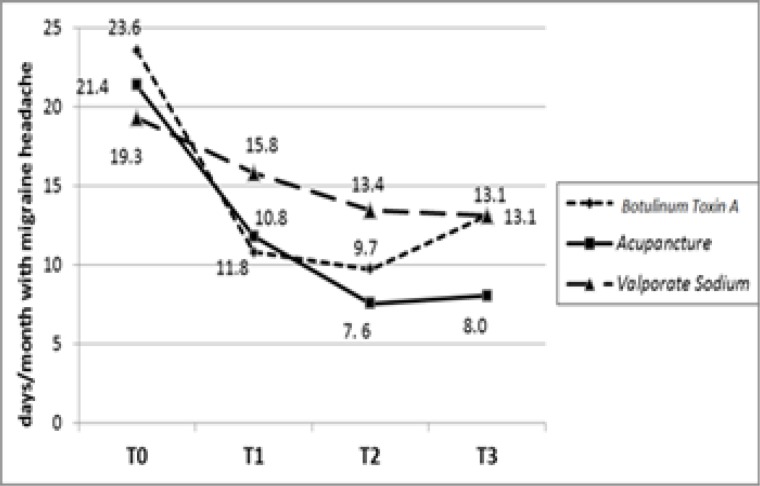
The trend of changes of mean numbers of days/month with migraine in groups treated with acupuncture (A) (P=0.0001), botulinum toxin A (B) (P=0.0001) and valproate sodium (C) (P=0.0001

The days/month with migraine significantly diminished from baseline to T3 in three groups (P=0.0001). To compare the groups, group A significantly had better results in decreasing days/month with migraine (P=0.0001). The differences of the numbers of days were insignificant at T0 (P=0.072) but were significant at T1 (P=0.0001) T2 (P=0.0001), and T3 (P=0.0001) (Figure 2).

**Figure 3:**
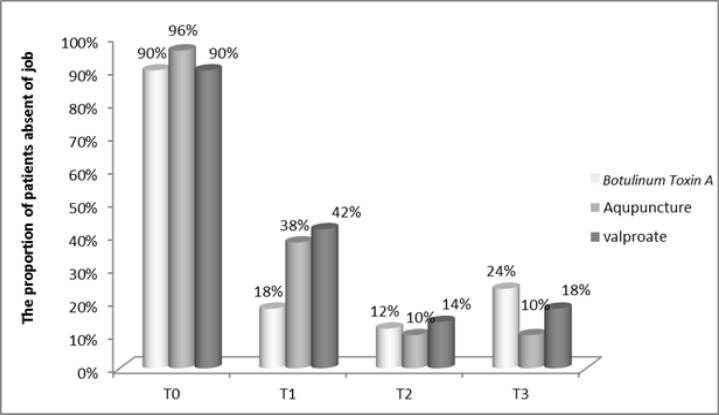
Comparison of the proportion of patients who made absence from work and social activities in groups treated with acupuncture (A), botulinum toxin A (B) and valproate sodium (C); T0:(P=0.443

The proportion of patients who missed out days due to headache significantly decreased in three groups at T1, T2, T3 (P=0.0001). The difference between groups was not significant at T0 (P=0.443), T2 (P=0.827) and T3 (P=0.179) but was significant at T1 (P=0.023) with less number of patients who were absent from work and social activities in botulinum toxin A group (figure 3).

**Figure 4:**
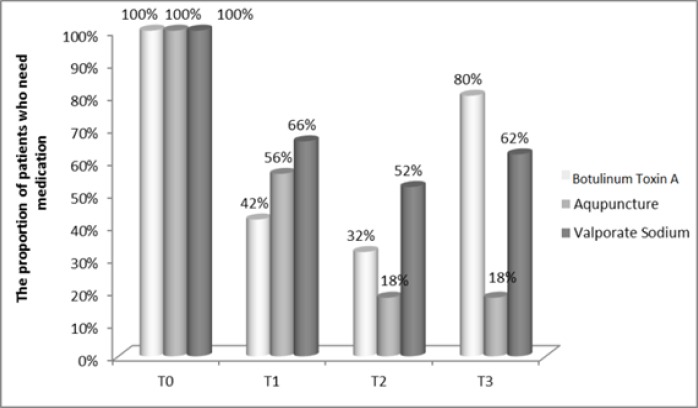
Comparison of the proportion of patients who need medications in groups treated with acupuncture (A), botulinum toxin A (B) and valproate sodium(C)

At admission time, all patients gave a medication history for their acute treatment of migraine attacks. After receiving these treatments, though the proportion of patients who need medications showed significant reduction, but the differences between the three groups were not significant till T1, followed by a significant difference at T2 (P=0.001) and T3 (P=0.0001) with a dramatic increase in drug use for acute treatment of headache in group B (figure 4).

**Figure 5:**
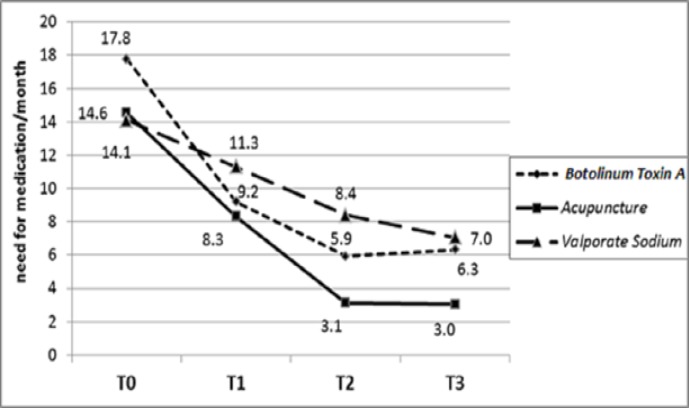
The trend of the changes of average number of times of needing medication per month in groups treated with acupuncture (A) (P=0.0001) and botulinum toxin A (B) (P=0.0001) and valproate sodium (C) (P=0.0001

The number of times of needing medication significantly diminished from baseline to T3 in three groups (P=0.001). To comparing the groups, group A significantly showed more reduction (P=0.0001). The differences were significant at baseline (P=0.006), T1 (P=0.0001), T2 (P=0.001) and T3 (P=0.0001) (figure 5) (table 2)

**Table 2:** The comparison of the changes of average number of times of needing medication per month in groups treated with acupuncture (A), botulinum toxin A (B) and valproate sodium(C)

**Measurement** ** time**	**Group**	**Mean±SD**	**P value**
**T0**	Botulinum toxin A	17.76±6.18	0.06
	Acupuncture	14.56±5.62	
	Valproate sodium	14.1±5.06	
**T1**	Botulinum toxin A	9.2±3.96	0.0001
	Acupuncture	8.32±4.52	
	Valproate sodium	11.3±5.43	
**T2**	Botulinum toxin A	5.92±3.78	0.0001
	Acupuncture	3.12±3.67	
	Valproate sodium	8.4±5.4	
**T3**	Botulinum toxin A	6.32±3.25	0.0001
	Acupuncture	3.34±3.96	
	Valproate sodium	7.04±4.32	

The number in each group=50

T0: baseline time,T1: one month after treatment,T2: two months after treatment, T3: three months after treatment

The incidence of nausea and vomiting among three groups has not differed with two months of follow-up (p>0.05), but after three months, the group that received botulinum toxin A showed a higher incidence of nausea and vomiting (P=0.027). The rate of side effects was significantly lower in group A than group B (6% vs. 22%) (P=0.021). The side effects of acupuncture treatment were only bleeding or subcutaneous hematoma formation and the adverse effects of botulinum toxin A included ptosis, facial masking or asymmetry. The reported side effects of sodium valproate after 3 months were asthenia in 5(10%), anorexia 2(4%), weight gain 2(4%), tremor 9(18%), somnolence 9(18%), insomnia 4(8%), alopecia 7(14%) of patients. 

## Discussion

Among the patients who completed this survey, the severity of headache based on VAS score diminished from baseline to T3 in the three groups and group A significantly showed more reduction. The same result has been achieved with regard to the days per month with migraine. The proportion of patients who missed out days due to headache significantly decreased in three groups at T1, T2, T3 with less number of patients who were absent from work and social activities in botulinum toxin A group. After receiving these treatments, they showed significant reduction in proportion of patients on medication during a three-time follow-up with a dramatic increase for medications in botulinum toxin A group at T3. The number of times a patient needs medication significantly diminished from baseline to T3 in three groups and group A significantly showed more reduction. 

In agreement with previous studies reporting encouraging results, we also found that the three methods are effective and safe with an extremely low complication rate (2, 15, 24-26). However, by comparing the three groups, the result of acupuncture group was better. Although several studies offer these interventions as choices of the prophylaxis of migraine especially in the patient unresponsive to previous treatments, but to the best of our knowledge, none of these studies have compared the two methods of acupuncture and Botulinum toxin-A injection. It might be the special feature of the present study but leads to some limitations comparing the results to similar studies. Thereby, confirming our results in group A, some other studies showed the effectiveness of acupuncture method in migraine, likewise our findings (23, 27-31). Moreover, other investigators demonstrated the therapeutic effects of botulinum toxin A injection for this purpose (26, 32-39). In Li Y. et al.’s (40) study acupuncture was performed twenty sessions per patient over a four-week period. In Linde K. et al.’s study (41) in the acupuncture group, the procedure was done for 12 sessions over 8 weeks. In Alecrim J. et al.’s study (42), the patients received 16 acupuncture sessions in 12 weeks. In these studies according to VAS score, days/month with migraine and the need for medications, the results showed no significant difference between treatment and placebo groups. The reason might be in part due to the number of treatment sessions. The other reason explaining the difference among the findings of studies could be the duration of follow-up time. The following two studies also found no significant difference between placebo and treatment groups, such that in Alecrim-Andrade J. et al.’s study (42) the follow- up time was six months and in Mathew N.T. et al’s. study (24) was nine months. If we followed-up our patients after three months, the results might be different. 

In general, the long lists of confirmed studies which cannot be discussed here, show a little difference among their results. The reason might be multifactorial and partly due to the different structures regarding population selection, study duration, number of treatment sessions and intervals. The other reason could be inner personal factors that show patient’s belief and trust about the effectiveness of the type of treatment. It is believed that the positive attitude towards a treatment method plays a powerful role in induced analgesia. Lack of awareness about these interventions makes accessibility difficult and this fear and anxiety could influence pain relief outcomes (12, 14). 

It is important to give the patients right and intelligent information and adequate explanation about this intervention. The next modulating factor might be the skill and experience of the interventionist and the chosen method for each patient. With regared to the adequate knowledge of anatomic variations for pain management, the physician is important in leading to effective injections at trigger point sites providing a more effective and cost saving way to perform these procedures. An individual may need more or less treatment sessions, since a course of a treatment varies due to several factors: the chronicity of disease, the patient’s general constitution and the body response to the treatment. One of our assisted outcomes was the adverse effects. As reported in literatures, there is a low percentage of unacceptable side effects in both groups that most of them are transient. The most common reported side effect in group A was on needle-insertion pain and in group B were the masked face and ptosis. Although both of these procedures appear to be safe and well tolerable to our patients, the complication rate was significantly lower in group A than group B. The reported side effects of sodium valproate after three months of treatment were asthenia, anorexia, weight gain, tremor, somnolence, insomnia, alopecia.

According to VAS, days/month with migraine pain, the need for medications and patient's absence from work and social activities revealed that the beneficial effects of botulinum toxin-A appear earlier than acupuncture which has a superior effectiveness. In this trial, it was revealed that both acupuncture and botulinum toxin A injection modalities can be added to the existing migraine headache therapy protocols. Nonetheless, the authors believe that, due to the short observation time, small size and the number of treatment sessions we may have presented preliminary results. This trial calls for future research studies dealing with this topic to clarify this issue, establish group of patients who are clear-responders or clear non-responders to these treatments, how long the beneficial effects last, what the unacceptable side effects are in long term, which treatment protocol leads to better results. Also, there are the other types of headache including cervicogenic headache which seems to respond well to these two kinds of treatment (43) and the one study suggests to compare them in treating sach headache. 

It is clearly known that Botox is generally effective during 3-6 months period and repetitive procedure is needed. If we had performed a long- term study, and performed repetitive procedure of Botox according to the protocol of PREEMPT I, the results could be different.

The strength of this study is the comparison of non-pharmacological treatments with a common used drug (sodium valproate) for migraine prophylaxis. But the nearly-weak point of this study is that, the investigators only evaluated the VAS score and the other measures such as of quality of life or disability, MIDAS, HIT-6 have not been assessed. Their assessment could have had noticeable results. Thus, repetition of such kind of study considering these measures is recommended to other interested investigators.

In conclusion** t**his trial demonstrates that acupuncture, botulinum toxin-A injection and pharmacological treatment have beneficial effects on chronic migraine; nevertheless, the result of acupuncture is more pronounced in comparison with botulinum toxin A injection and sodium valproate. As a result, acupuncture may be the preferred method because of more effectiveness and less side effects.
